# Pilot study of decision support tools on breast cancer chemoprevention for high-risk women and healthcare providers in the primary care setting

**DOI:** 10.1186/s12911-018-0716-5

**Published:** 2018-12-17

**Authors:** Rita Kukafka, Jiaqi Fang, Alejandro Vanegas, Thomas Silverman, Katherine D. Crew

**Affiliations:** 1 Columbia University, Mailman School of Public Health, 622 West 168th Street, PH-20, New York, NY 10032 USA; 2 Columbia University, College of Physicians and Surgeons, New York, NY USA; 3 Herbert Irving Comprehensive Cancer Center, New York, NY USA

**Keywords:** Chemoprevention, Breast cancer, Decision support, Decision conflict, Risk communication, Decision aids

## Abstract

**Background:**

Breast cancer chemoprevention can reduce breast cancer incidence in high-risk women; however, chemoprevention is underutilized in the primary care setting. We conducted a pilot study of decision support tools among high-risk women and their primary care providers (PCPs).

**Methods:**

The intervention included a decision aid (DA) for high-risk women, *RealRisks*, and a provider-centered tool, Breast Cancer Risk Navigation (*BNAV)*. Patients completed validated surveys at baseline, after *RealRisks* and after their PCP clinical encounter or at 6-months. Referral for high-risk consultation and chemoprevention uptake were assessed via the electronic health record. The primary endpoint was accuracy of breast cancer risk perception at 6-months.

**Results:**

Among 40 evaluable high-risk women, median age was 64.5 years and median 5-year breast cancer risk was 2.19%. After exposure to *RealRisks*, patients demonstrated an improvement in accurate breast cancer risk perceptions (*p* = 0.02), an increase in chemoprevention knowledge (*p* < 0.01), and 24% expressed interest in taking chemoprevention. Three women had a high-risk referral, and no one initiated chemoprevention. Decisional conflict significantly increased from after exposure to *RealRisks* to after their clinical encounter or at 6-months (*p* < 0.01). Accurate breast cancer risk perceptions improved and was sustained at 6-months or after clinical encounters. We discuss the side effect profile of chemoprevention and the care pathway when *RealRisks* was introduced to understand why patients experienced increased decision conflict.

**Conclusion:**

Future interventions should carefully link the use of a DA more proximally to the clinical encounter, investigate timed measurements of decision conflict and improve risk communication, shared decision making, and chemoprevention education for PCPs. Additional work remains to better understand the impact of decision aids targeting both patients and providers.

**Trial registration:**

ClinicalTrials.gov Identifier: NCT02954900 November 4, 2016 Retrospectively registered.

## Background

Breast cancer is the most common cancer among women in the United States, with an estimated 1 in 8 women developing invasive breast cancer during her lifetime [1]. More than 252,700 new cases and 40,610 deaths due to breast cancer are expected to occur among U.S. women in 2017 [2]. Women with a 5-year invasive breast cancer risk greater than 1.67% or a lifetime risk greater than 20% based upon the Gail model [3] have the option of taking a chemopreventive medication. Chemoprevention with selective estrogen receptor modulators (SERMs) and aromatase inhibitors (AIs) has been shown to reduce invasive breast cancer risk by up to 50–65% among high-risk women in randomized controlled trials [4–7]. Grounded in strong evidence, the U.S. Preventive Services Task Force (USPSTF), National Comprehensive Cancer Network (NCCN), American Society for Clinical Oncology (ASCO), and the National Institute for Health and Care Excellence (NICE) recommend that clinicians discuss preventive therapy with high-risk women [8–11]. However, uptake of SERMs or AIs for the prevention of breast cancer is extremely low [12]. In a meta-analysis of therapeutic agent uptake to prevent breast cancer among women at increased risk, Smith et al. found that uptake was 25.2% among women screened for clinical trials, but only 8.7% (95% CI, 6.8–10.9) in non-trial settings [13].

Decision aids can help to improve communication between providers and patients, and can assist patients clarify how important the potential benefits and harms are to them. However, studies that examined the effect of decision aids on SERM use found that while knowledge about risks and benefits increased, decisions were rarely influenced. *Guide to Decide*, a decision aid that informed high-risk postmenopausal women about potential chemoprevention benefits and side effects resulted in no tamoxifen uptake and only 0.5% uptake of raloxifene [14]. The *Ready, Set, GO GAIL!* study involved PCPs using the Gail model to screen more than 5700 women [15]. Although 868 (15.2%) women were classified as high-risk for breast cancer, only 14.7% were referred for risk counseling, 6.4% attended the consultation, and 2% started chemoprevention. The *Breast*CARE randomized controlled trial revealed that more women were referred for high-risk consultation in the intervention group compared to controls, however there was limited communication about chemoprevention documented in the medical record [16].

The literature is surprisingly scant on decision aids targeting both patients and providers [17], and most studies on chemoprevention decision-making have been based on hypothetical scenarios to evaluate levels of interest, which may ineffectively predict actual uptake [12]. Studies confirm the importance of the primary care provider recommendation for the decision to take a SERM for breast cancer risk reduction [18–20]. However, this recommendation was more likely to be followed when SERM use discussions assess patient attitudes toward medication and relate to those when discussing chemoprevention options to make the information relevant to the patients [18]. Since breast cancer chemoprevention is not generally diffused in the primary care setting, more effective tools are needed to inform both providers and patients about the risks and benefits of SERMs and AIs, help them to identify available options and deliberate those options in light of patients values and preferences.

In this paper, we report results of a pilot study conducted to examine the efficacy of two decision support tools, the *RealRisks* decision aid (DA) for patients and the Breast cancer risk NAVigation (*BNAV*) decision support tool for primary care providers (PCPs) [21]. Providers received *BNAV* decision support at the time their patient completed *RealRisks*, thus the tools when integrated into clinical workflow were intended to complement one another. We sought to determine whether our decision support tools increase patient’s accuracy of breast cancer risk perceptions, and breast cancer and chemoprevention knowledge. We also sought to identify referrals for consultation at a high-risk breast clinic, and chemoprevention uptake among women at high risk for breast cancer post intervention.

## Methods

### Study population and eligibility criteria

**Fig. 1:**
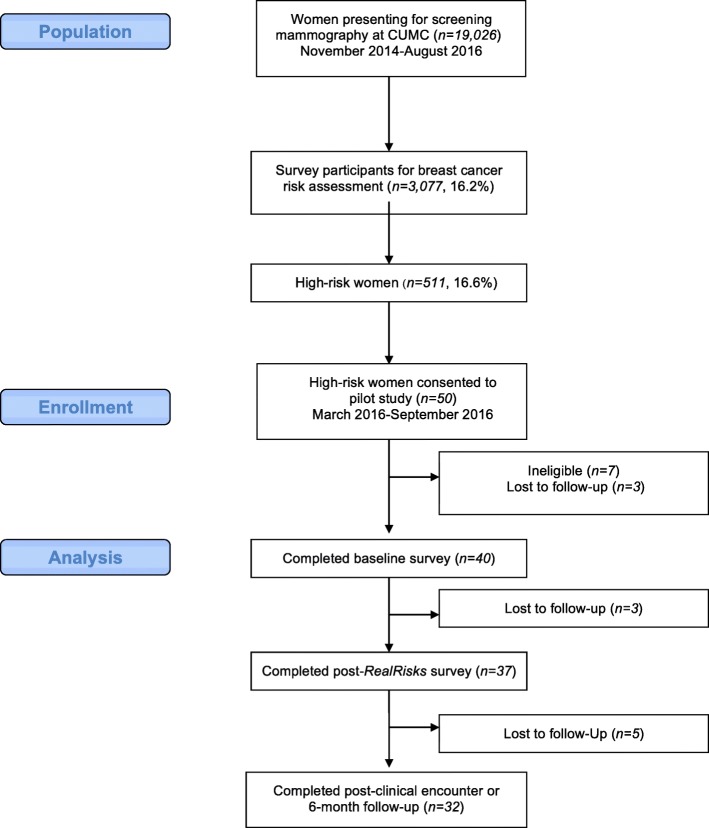
Consort Diagram

Women (*N* = 19,026) presented for screening mammography at Columbia University Medical Center (CUMC) in New York, NY between 2014 and 2016 (Fig. 1) [22]. Among them, 3743 (19.7%) were approached for enrollment into the Know Your Risk: Assessment at Screening (KYRAS) for breast cancer study, and 3077 (82.2%) consented and completed a baseline survey on demographics and breast cancer risk factors. Of the 3077, 511 (16.6%) women were identified as high-risk for breast cancer according to the Gail model. These high-risk women were informed of their breast cancer risk status and given a brochure to the CUMC breast clinic for a high-risk consultation. A subset of participants (*N* = 50) from this larger study was recruited into a single-arm pilot intervention study of the chemoprevention decision support tools. Eligibility criteria for the pilot included the following: 1) women, age 35–75 years; 2) 5-year invasive breast cancer risk ≥1.67% based upon the Gail model [3]; 3) having a primary care provider (PCP) at CUMC; 4) English or Spanish-speaking. Those with any history of breast cancer or with current or prior use of a selective estrogen receptor modulator (SERM) or aromatase inhibitor (AI) for breast cancer risk reduction were excluded. Having a PCP at CUMC was an inclusion criteria to ensure that both patient and provider would have access to the decision support tools and we could access the electronic health record at CUMC. Providers from primary care clinics at CUMC were invited to participate if they had an identified patient eligible for breast cancer chemoprevention. This study was approved by the institutional review board at CUMC and registered at clinicaltrials.gov (NCT02954900).

### Study interventions

**Fig. 2:**
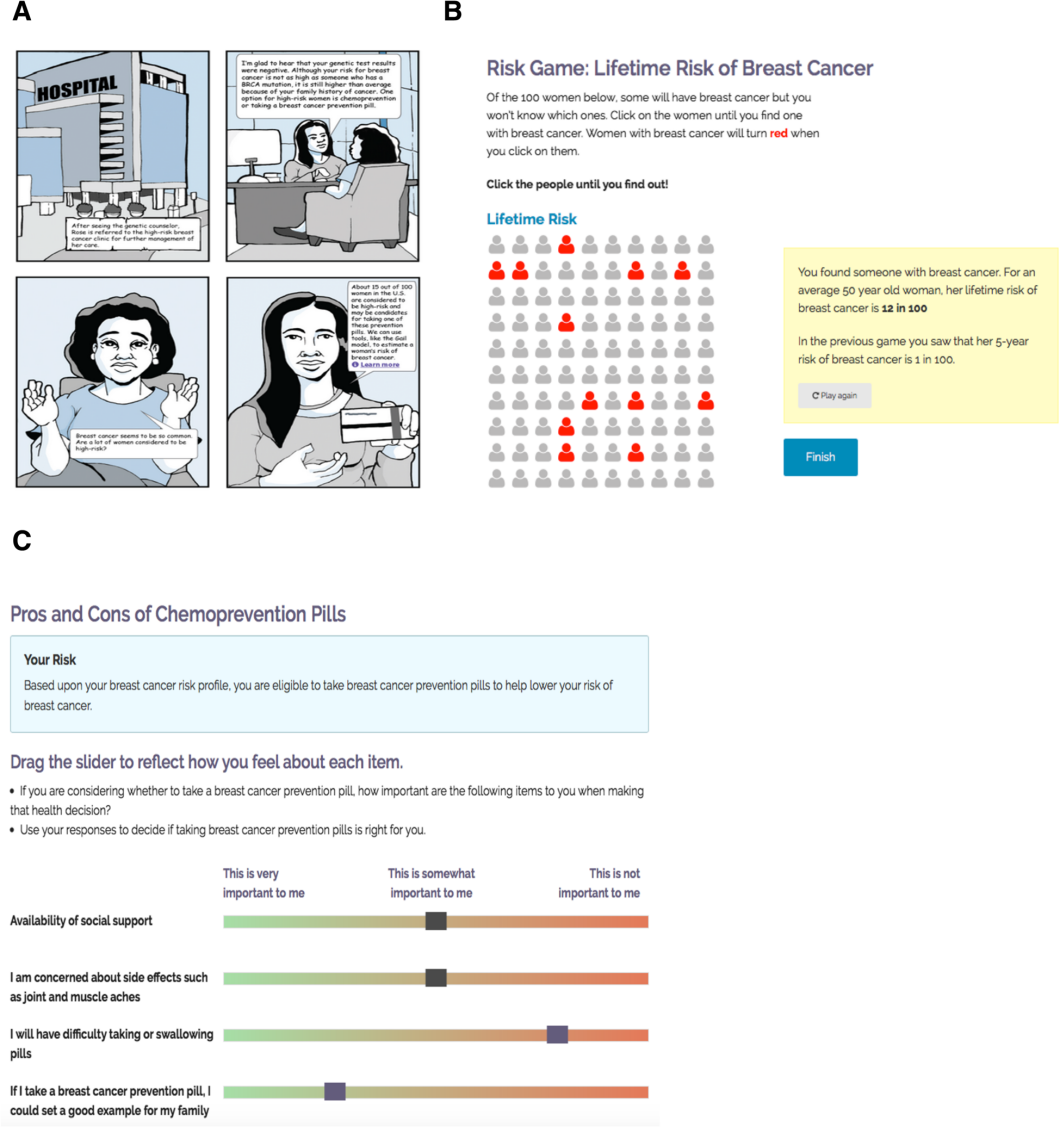
Screenshots of *RealRisks*, patient-centered decision aid: (**a**) Graphic novel-style narrative; (**b**) Interactive game to communicate breast cancer risk; (**c**) Preference elicitation about the risks and benefits of chemoprevention

*RealRisks* is a web-based patient-centered decision aid (DA) designed to increase the following: 1) accurate breast cancer risk perceptions; 2) breast cancer and chemoprevention knowledge; and 3) self-efficacy to engage in a collaborative dialogue about breast cancer risk and chemoprevention decisions (Fig. 2). We designed *RealRisks* by involving both patients and providers in multiple design sessions and usability studies to arrive at guiding principles that focused on the following: 1) options for providing information of breast cancer risk and chemoprevention, 2) “interactive games” to communicate breast cancer risk, and 3) patient preference elicitation to weigh the risks and benefits of SERMs and AIs (Fig. 2). An early prototype was evaluated in focus groups among women of various ethnicities from New York City. In this evaluation, accuracy of breast cancer risk perception (perceived minus actual breast cancer risk according to the Gail model [3]) significantly improved after interacting with *RealRisks*, even in the subgroup of women with low numeracy [23]. After the initial prototype was developed, we conducted usability studies with English- and Spanish-speaking women to determine how they navigated, engaged with and understood the information in *RealRisks* [21]. Using surveys, think-aloud protocols, and subject recordings, we identified several themes relating to the usability of *RealRisks*, specifically in the content, ease of use, and navigability of the application. By conducting studies in two languages with a diverse multi-ethnic population, we were able to implement interface changes to make *RealRisks* accessible to users with varying levels of health literacy and acculturation.

*RealRisks* includes the option to view the material in a text-heavy, or “information dense,” format or in an “information light” format that primarily uses pictures. The DA also has an audio option and Spanish translations. The educational modules include: 1) Breast cancer risk (breast cancer risk factors, calculation of personal breast cancer risk according to the Gail model, interactive games on risk communication); and 2) Chemoprevention (what is chemoprevention, risks and benefits of SERMs and AIs for chemoprevention, preference elicitation of chemoprevention). Through the *RealRisks* DA, we collect information on breast cancer risk factors to calculate a patient’s Gail risk score. We also collect factors that influence the decision making about chemoprevention through the preference elicitation game. *RealRisks* generates an action plan for patients summarizing their personalized breast cancer risk profile and preferences for chemoprevention. A provider view of the action plan, which is much shorter in length and designed to be actionable, is also made available to the provider through *BNAV*.

**Fig. 3:**
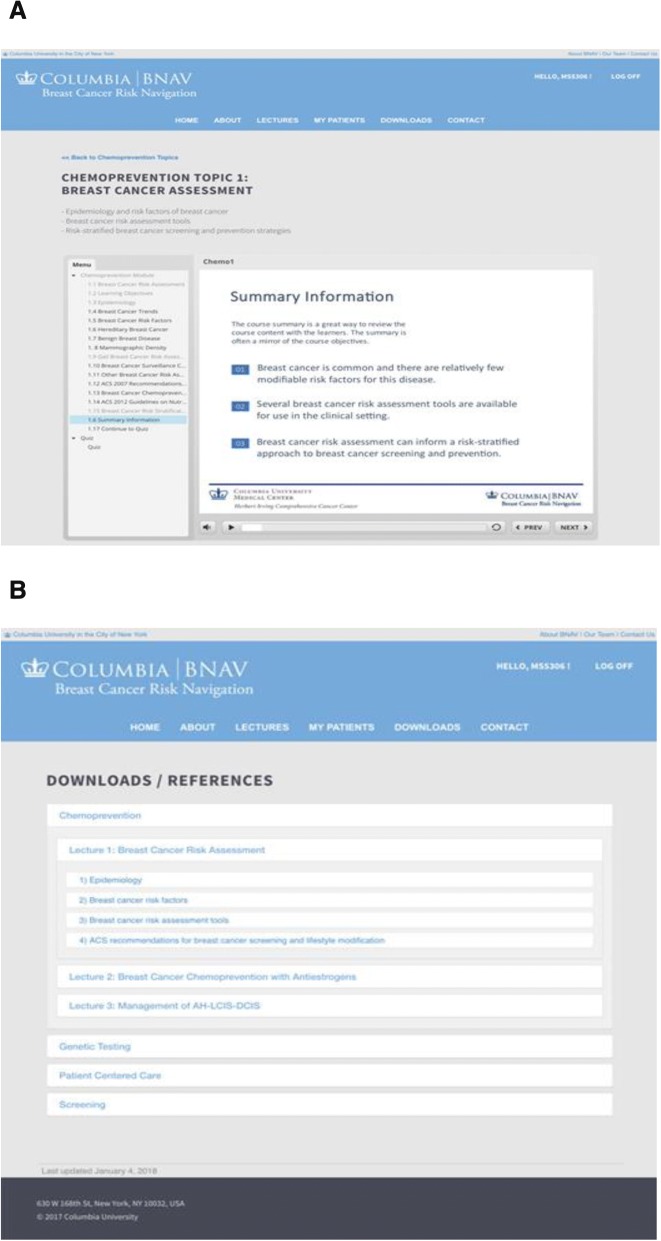
Screenshots of *BNAV* (Breast cancer risk NAVigation) provider-centered tool: (**a**) Slide presentations; (**b**) Link to download references

The provider-facing *BNAV* tool (Fig. 3) uses a two-pronged strategy to improve knowledge among healthcare providers about breast cancer risk assessment and chemoprevention. After patients complete *RealRisks*, their providers receive the tailored action plan via secure health messaging and are offered access the web-based *BNAV* toolbox. Based on the Theory of Planned Behavior [24], the toolbox contains a collection of information and resources that includes: 1) up-to-date guidelines and interactive educational presentations (attitudes); 2) video testimonials from experts in the field and a social component that enables a provider to compare his or her performance against aggregate, anonymous data of his or her peers (subjective norm); and 3) a repository of their patients’ breast cancer risk, together with the action plans generated by the patients’ interactions with *RealRisks* (perceived behavioral control). The *BNAV* chemoprevention module includes resources on breast cancer risk assessment, benefits and risks of chemoprevention, and how to manage the side effects of SERMs and AIs. Over time, other modules have been integrated into *RealRisks* (genetic testing for hereditary breast and ovarian cancer syndrome (HBOC), screening recommendations and guidelines, and risky health behaviors and lifestyle modification) and *BNAV* (genetic testing for HBOC, screening recommendations and guidelines, and patient-centered care), which allow providers to self-direct viewing resources outside of the clinical encounter. Each module takes about 10–20 min to view and can be completed during multiple sittings. To evaluate *BNAV*, individual interviews were conducted with 10 PCPs [25]. We found that few providers routinely used breast cancer risk calculators in their practice and they expressed concerns about the added burden of incorporating these tools into the clinic visit and being unfamiliar with chemoprevention.

### Outcome measures and data collection

High-risk women completed self-administered questionnaires at baseline, immediately after completing *RealRisks*, and 6 months after baseline or after the clinical encounter with their PCP. Health literacy, subjective and objective numeracy, and acculturation were assessed at baseline using brief validated measures of each construct [26, 27]. Breast cancer and chemoprevention knowledge was assessed using a 13-item scale, with adequate knowledge defined as at least 50% correct responses [28]. A participant’s confidence in communicating with her PCP was measured as an average of three items (range: 0–100%) [29]. Breast cancer risk perception was assessed by four items of absolute estimate, comparative risk assessed on a 3-point Likert scale, and numeric 5-year and lifetime risk on a scale of 0 to 100% [30]. Breast cancer worry was assessed using responses to two questions on a 7-point Likert scale [31, 32]. Self-efficacy was measured as an average of 5 items to assess perceived confidence in making a choice (range: 0–100%) [33]. Decision conflict was measured using the low literacy version of the Decision Conflict Scale (DCS) after exposure to *RealRisks* and at 6 months or after the PCP clinical encounter. The total score was calculated by averaging the 10 individual item scores so that higher scores indicated higher decisional conflict (range: 0–100) [33]. Scores lower than 25 on the scale are associated with implementing decisions whereas scores greater than 37.5 indicate high decision conflict, which is characterized by decision delay and/or uncertainty. Subscores of the scale that focus on factors contributing to uncertainty (e.g.*,* feeling uncertain, uninformed, unclear about values, and unsupported in decision-making) were also reported on a range from 0 to 100 [33]. Behavioral intent for chemoprevention was assessed as previously described [14]. Referral to the high-risk breast clinic and chemoprevention uptake were assessed at 6 months via the electronic health record (EHR).

### Statistical analysis

Our primary endpoint was accuracy of perceived breast cancer risk, defined as perceived lifetime risk within ±10% of actual lifetime risk according to the Gail model, at 6 months compared to baseline. The 10% range on either side of the Gail model risk estimate has been commonly used to provide a reasonable margin within which responses are labeled as accurate [34–37].McNemar’s test was used to compare accurate breast cancer risk perception and adequate breast cancer and chemoprevention knowledge at baseline and follow-up. Paired t-tests were utilized to assess changes in continuous measures of differences in perceived and actual lifetime breast cancer risk, breast cancer and chemoprevention knowledge, breast cancer worry, self-efficacy, and decision conflict from baseline to post-*RealRisks*, from baseline to post-clinical encounter or 6 months, and from post-*RealRisks* to post-clinical encounter or 6 months. All analyses were conducted using SAS version 9.4 (Cary, NC) and a *p*-value < 0.05 was considered to be statistically significant.

## Results

### Participants

Table 1 summarizes the baseline characteristics of the forty women who met all eligibility criteria and completed the baseline survey. The majority (95%) were postmenopausal women with a median age of 64.5 years (range, 49–72 years). Our sample was racially and ethnically diverse with 42.5% Hispanics, 40.0% non-Hispanic white, 12.5% non-Hispanic black and 5.0% Asian or other. The mean 5-year invasive breast cancer risk, based on the Gail model, was 2.38 ± 0.81%. Thirty-five percent reported having benign breast disease and half of them had a first-degree family history of breast cancer. Compared to non-Hispanic women, Hispanics were less educated and had lower acculturation, health literacy, and numeracy.

**Table 1:** Baseline characteristics of high-risk women identified during screening mammography, Columbia University Medical Center (CUMC), New York, NY (Mar 2016-Sept 2016) stratified by Hispanic and non-Hispanic ethnicity

Patient Characteristics	Hispanics (*N* = 17)	Non-Hispanics (*N* = 23)	Total (*N* = 40)	*p*-value
Age, years, N (%)				0.57
< 50	2 (11.8)	0	2 (5.0)	
50–59	1 (5.9)	4 (17.4)	5 (12.5)	
60–69	10 (58.8)	14 (60.9)	24 (60.0)	
> 70	4 (23.5)	5 (21.7)	9 (22.5)	
Median (range)	64 (49–72)	66 (50–72)	64.5 (49–72)	
Mean (SD)	63.1 (7.2)	64.3 (6.5)	63.8 (6.7)	
Menopausal status, N (%)				0.17
Premenopausal/Perimenopausal	2 (11.8)	0	2 (5.0)	
Postmenopausal	15 (88.2)	23 (100.0)	38 (95.0)	
Race/ethnicity, N (%)				N/A
Hispanic	17 (100.0)	0	17 (42.5)	
Non-Hispanic white	0	16 (69.6)	16 (40.0)	
Non-Hispanic black	0	5 (21.7)	5 (12.5)	
Other	0	2 (8.7)	2 (5.0)	
Education level, N (%)				< 0.01
High school or less	11 (64.7)	1 (4.3)	12 (30.0)	
Some college or bachelors	4 (23.5)	11 (47.8)	15 (37.5)	
Graduate or professional degree	2 (11.8)	11 (47.8)	13 (32.5)	
Benign breast disease, N (%)	7 (41.2)	7 (30.4)	14 (35.0)	0.75
First-degree family history of breast cancer, N (%)	12 (70.6)	8 (34.8)	20 (50.0)	0.05
5-year invasive breast cancer risk, %				0.45
Median (range)	2.01 (1.68–5.64)	2.23 (1.70–4.55)	2.19 (1.68–5.64)	
Mean (SD)	2.26 (0.92)	2.46 (0.72)	2.38 (0.81)	
Lifetime invasive breast cancer risk, %				0.57
Median (range)	7.87 (5.25–23.85)	8.85 (4.42–14.14)	8.71 (4.42–23.85)	
Mean (SD)	9.88 (4.99)	9.10 (2.97)	9.43 (3.92)	
Age of menarche, N (%)				0.02
7–11 years	1 (5.9)	8 (34.8)	9 (22.5)	
12–13 years	11 (64.7)	14 (60.9)	25 (62.5)	
14+ years	5 (29.4)	1 (4.3)	6 (15.0)	
Age of first birth, N (%)				0.17
No births	1 (5.9)	2 (8.7)	3 (7.5)	
< 20 years	4 (23.5)	4 (17.4)	8 (20.0)	
20–24 years	4 (23.5)	3 (13.0)	7 (17.5)	
25–29 years	4 (23.5)	1 (4.3)	5 (12.5)	
30+ years	4 (23.5)	13 (56.5)	17 (42.5)	
Hormone replacement therapy use	2 (11.8)	4 (17.4)	6 (15.0)	0.93
Mean acculturation (SD) [range, 1–5]	1.36 (0.56)	4.89 (0.26)	3.40 (1.81)	<.01
Adequate health literacy, N (%)	11 (64.7)	22 (95.7)	33 (82.5)	0.03
Mean subjective numeracy (SD) [range, 1–6]	3.22 (0.19)	4.54 (0.67)	4.05 (0.95)	<.01
Adequate numeracy, N (%)	6 (35.3)	19 (82.6)	25 (62.5)	<.01
Mean Confidence (SD) [range, 1–10]	9.48 (0.87)	8.95 (1.61)	9.18 (1.36)	0.20

### Patient reported outcomes at baseline, post-intervention and 6 months.

Table 2 summarizes the patient-reported outcomes at baseline, post-intervention (*RealRisks*) and at 6 months or after the clinical encounter with their primary care provider (PCP). At baseline, the majority of patients (75%) overestimated their perceived risk of breast cancer. Accurate breast cancer risk perceptions increased from 38% at baseline to 63% at 6 months (*p* = 0.03). The mean difference between perceived and actual lifetime breast cancer risk according to the Gail model decreased from 23.7% (SD 24.7) at baseline to 12.1% (SD 18.1) post-*RealRisks* (*p* = 0.01). After exposure to *RealRisks*, there was no significant change in breast cancer knowledge; however, mean chemoprevention knowledge scores significantly improved from baseline to follow-up, but this difference diminished over time (0.71 at baseline, 3.69 post-*RealRisks*, and 1.94 at 6 months, *p* < 0.01 for both comparisons). Breast cancer worry and self-efficacy in chemoprevention decision making did not change significantly. However, decision conflict increased from post-intervention to 6 months (17.92 vs. 43.44, *p* < .01). After exposure to *RealRisks*, 23 women (64%) experienced some or no decisional conflict with scores below 25 compared with 9 women (28%) at 6 months. Sub-scores for every decisional conflict domain were in the same direction, with significantly higher scores at 6 months compared to post-intervention. In terms of chemoprevention intention, 24% of participants expressed interest in taking chemoprevention, 47% were unsure, and 29% were not interested. Three (7.5%) women were referred for high-risk consultation and none had initiated chemoprevention at 6 months. There were no significant differences in patient-reported outcomes between Hispanic and non-Hispanic women, except that Hispanic women tended to have higher mean breast cancer worry scores (4.72 vs. 2.09 at baseline, 5.02 vs. 2.50 post-*RealRisks*, and 4.82 vs. 2.11 at 6 months, respectively).

**Table 2:** Patient-reported outcomes at baseline, post-intervention (*RealRisks*) and at 6 months or after the clinical encounter with their primary care providers

Patient Outcome Measures	Baseline (T0)	Post-*RealRisks* (T1)	Post-Clinical Encounter or 6 months (T2)	*p*-value		
	*(N = 40)*	*(N = 37)*	*(N = 32)*	T0-T1	T0-T2	T1-T2
Breast Cancer Risk Perception						
Mean difference between perceived and actual lifetime breast cancer risk (SD)	23.67 (24.68)	12.09 (18.13)	20.35 (27.65)	0.01	0.36	
Accurate breast cancer risk perception, N(%)	15 (39.47)	23 (63.89)	20 (62.50)	0.02	0.02	
Breast cancer knowledge						
Mean number correct (SD) [range, 0–15]	8.00 (1.69)	8.59 (1.38)	7.94 (1.90)	0.10	0.14	
Adequate knowledge, N (%)	25 (64.10)	30 (81.08)	17 (53.13)	0.13	.32	
Chemoprevention knowledge						
Mean number correct (SD) [range, 0–8]	0.71 (1.46)	3.69 (2.39)	1.94 (1.92)	<.01	<.01	
Adequate knowledge, N (%)	4 (10.53)	18 (50.00)	9 (28.13)	< 0.01	0.10	
Mean breast cancer worry (SD) [range, 1–7]	3.17 (1.80)	3.66 (2.17)	3.30 (2.06)	0.17	0.75	
Mean self-efficacy in chemoprevention (SD) [range, 0–100]	56 (15.81)	60.22 (15.45)		0.24		
Mean decision conflict (SD) [range, 0–100]		17.92 (19.51)	43.44 (31.30)			<.01
Decision conflict, N (%)						<.01
Decision implementation (< 25)		23 (63.9)	9 (28.1)			
Unsure about implementation (25–37.5)		5 (13.9)	5 (15.6)			
Decision delay (> 37.5)		8 (22.2)	18 (56.3)			
Decision conflict sub-scores (SD) (range, 0–100)						
Informed subscore		9.03 (12.66)	24.48 (19.74)			<.01
Values clarity subscore		8.33 (15.24)	23.44 (20.02)			<.01
Support subscore		7.87 (9.33)	17.71 (18.66)			0.01
Chemoprevention intention, *N* (%)						
Yes		9 (24.3)				
No		12 (32.4)				
Unsure		17 (45.9)				
High-risk referrals, *N* (%)			3 (7.5)			
Chemoprevention uptake, *N* (%) 0						

## Discussion

Our intervention demonstrated a significant improvement in accuracy of breast cancer risk perception and an increase in chemoprevention knowledge. It was notable that accuracy of breast cancer risk perceptions significantly improved post *RealRisks*, and was sustained at 6 months. The finding that decision conflict significantly increased from post-intervention to 6 months or after clinical encounters with PCPs was unexpected. The literature is not clear on why the decision to accept or reject chemoprevention would become more difficult with the passage of time. Possible explanations may reside with the decision itself, the care pathway when the DA was introduced, or a combination of both.

Decision conflict is not simply recognition that advantages and disadvantages exist for any given option. It is also an undesirable state of discomfort and internal conflict experienced when facing a difficult decision [38]. The decision to take chemoprevention can raise an undesirable state of discomfort given the perception that SERMs or AIs are “cancer drugs” and tradeoffs between the risks and benefits of these medications. It has been proposed that the side effect profile of chemoprevention medications, coupled with wide-ranging concerns about the emotional impact of taking a medication, leads to medication avoidance. Specifically, women might decide to avoid chemoprevention because of the affect-laden responses associated with the term “side effect” and their belief that these medications will increase, rather than decrease, their level of health-related stress [39]. [40–42] Concerns about possible side effects, such as uterine cancer, thromboembolism, and menopausal symptoms, are the primary reasons why women are reluctant to start breast cancer chemoprevention [43–49]. This may also explain results found in previous studies that improved knowledge may result in women becoming more reluctant to take medications that are associated with potentially harmful health risks [14, 50].

It is recognized that negative emotions experienced while making a choice involving difficult tradeoffs can potentially impact decision conflict over time. The judgment and decision-making literature suggests that people can react to emotionally laden decisions by altering the amount or content of thought about the decision (emotion-focused coping). This can result in avoidant behaviors, for example declining to make a decision [51], allowing another make the decision for you, or exhibiting an increased tolerance for the status quo option [52]. Studies suggest that decision makers are likely to face between attribute tradeoffs required by decision conflict when the attributes are relatively low in emotional tradeoff difficulty. Conversely, they tend to elude these tradeoffs when attributes are higher in emotional tradeoff difficulty. Thus, it is plausible that increased decision conflict from post intervention to 6 months was due to decision avoidance, given that the decision to take chemoprevention requires women to evaluate a number of emotionally laden tradeoffs, mainly between the potential benefits and perceived barriers to chemoprevention medication uptake [53].

As previously asserted in the literature, it is mostly acceptable for decisional conflict to be high if measured shortly after options have been presented, however after the patient has been given the opportunity to incorporate their preferences into the presented options and make a decision, decisional conflict should be low [54]. An equally plausible assertion is that for a patient who prudently weighs competing options decision conflict will be high and even with the passage of time, a rational patient would state that the decision was difficult [55, 56]. Few randomized controlled trials have investigated timed measurements of decision conflict, particularly in underserved populations. Our conclusions are limited due to study design and the lack of a control group in our pilot study, however this area warrants further investigation.

With respect to care pathway, although we tried to integrate the web-based patient and provider decision support tools into clinic workflow, use of the *RealRisks* DA was not closely linked with the PCP clinical encounter. Many of the patient-reported outcomes, such as accurate breast cancer risk perceptions and chemoprevention knowledge, diminished from immediately post-intervention to 6 months or after the clinical encounter. The increase in decision conflict may also reflect a diminished patient outcome since significantly fewer women were in the decision implementation phase according to the DCS. Nearly a quarter of women expressed interest in chemoprevention uptake after completing *RealRisks*, yet few women were referred for high-risk consultations and none of the participants had initiated chemoprevention at 6 months. Perhaps linking use of the DA more proximally to the clinical encounter will improve our patient reported outcomes. In future work, we have incorporated alerts and other cues to remind the patient to complete the *RealRisks* DA within a week prior to the clinical encounter.

Similar to previous studies, our intervention demonstrated that decision aids improve knowledge in those who use them. However, also like previous studies, increased knowledge does not lead to increased chemoprevention uptake for the purpose of reducing breast cancer risk. Improved accuracy of risk perceptions is an important measure of the quality of a decision aid [57], and while this is a prerequisite to informed decision making, it is difficult to know if this results in clinically useful decision making. Even when breast cancer risk seems to be understood, willingness to take chemoprevention medication remains low among women who are identified as eligible based on their Gail risk score [14, 58]. Previous research has demonstrated that health decision making may be based on heuristics and feelings, rather than on an accurate understanding of risk information [59, 60]. As such, individuals may not always process and act upon the risk information presented to them in the ways that healthcare providers intend. In addition to precise probabilistic risk information, lived experiences and particularly individual experiences with cancer have been shown to influence chemoprevention decisions [58, 61].

Several studies have demonstrated that recommendations from physicians and effective communication greatly affect patients’ decision making in chemoprevention uptake [43]. Based upon data from key informant interviews, we found that PCPs reveal unfamiliarity with breast cancer risk assessment tools such as the Gail Model and a lack of confidence in prescribing chemoprevention. PCPs also reveal a preference to refer their patients to specialists for consultation about breast cancer risk reduction options, which may imply that the they were not well-informed about breast cancer preventive strategies available to patients [25]. Although all high-risk women and PCPs were given information on the CUMC breast clinic for high-risk consultations, it appears this was insufficient to alter practice patterns for most PCPs.

Trials of decision support tools designed to increase uptake of breast cancer chemoprevention targeting both patients and providers have been limited. Uptake remains low [14], [15] and as demonstrated in a randomized controlled trial of the *BreastCARE* intervention discussions about chemoprevention were still limited [16]. This prior literature suggests that just targeting high-risk women or PCPs alone is ineffective. Additional work remains to better understand the impact of decision aids targeting both patients and providers.

### Limitations

Limitations of our study include the lack of a concurrent control arm, the relatively small sample size, and conducting the study in an urban academic center with access to a high-risk clinic, all of which limit the generalizability of our findings. In addition, although we found no significant differences in age, race, and racial distribution between the pilot sample and KYRAS high-risk patients, our sample was self-selected from the larger KYRAS screening study. The study sample was older than what we expected and may not be totally representative of a higher risk younger population who are likely more appropriate for chemoprevention and the *RealRisks* DA. Additionally, we had higher than anticipated loss to follow-up of about 20%. Our short-term follow-up of 6 months may have been insufficient to assess clinical outcomes such as high-risk clinic referrals and actual chemoprevention uptake.

## Conclusions

We developed decision support tools for both patients and their PCPs, which include personalized risk reports and education about breast cancer risk and chemoprevention. Our study population was racially and ethnically diverse and our patient-centered DA, which is available in English and Spanish, was rigorously tested in women of multiple ethnicities with varying levels of health literacy and numeracy. In addition to using validated outcome measures, we were able to assess referrals to the high risk breast clinic and actual chemoprevention uptake using electronic health records.

The results of our initial pilot study have informed the design and conduct of a larger randomized controlled trial of 300 high-risk women assigned to standard educational materials alone or in combination with *RealRisks* and *BNAV* (NCT03069742). We will target younger, healthier women with higher breast cancer risk, including those with high-risk benign breast lesions such as atypical hyperplasia and lobular carcinoma in situ. These women are likely to derive a greater benefit from breast cancer risk reduction and a lower risk of serious side effects. To reinforce use of the patient-centered *RealRisks* DA, we will set up automated reminders to revisit the tool prior to their next PCP clinical encounter, next screening mammography visit, and birthday (as breast cancer risk increases with advancing age). We will enhance provider engagement with an enhanced *BNAV* tool, which will offer continuing medical education (CME) credit and additional modules on breast cancer screening and other topics relevant to PCPs. We have already developed modules on risk communication and shared decision making. We will elicit patient preferences for chemoprevention, specifically factors that are most important and least important to chemoprevention decisions, which we will summarize for providers prior to the clinical encounter. Even if we do not observe a significant increase in chemoprevention uptake with the addition of *RealRisks* and *BNAV* compared to standard educational materials, our goal is to also increase informed choice, decrease decision conflict, and facilitate SDM during the clinical encounter. Facilitating discussions about breast cancer chemoprevention between clinicians and high-risk women is in accordance with recommendations from the U.S. Preventive Services Task Force (USPSTF), American Society for Clinical Oncology (ASCO), National Comprehensive Cancer Network (NCCN), and the National Institute for Health and Care Excellence (NICE) [8–11].

## Abbreviations

AIs: Aromatase inhibitors
ASCO: American Society for Clinical Oncology
*BNAV*: Breast cancer risk NAVigation decision support tool
DA: Decision aid
HBOC: Hereditary breast and ovarian cancer syndrome
NCCN: National Comprehensive Cancer Network (NCCN)
NICE: National Institute for Health and Care Excellence
PCPs: Primary care providers
SDM: Shared decision-making
SERMS: Selective estrogen receptor modulators
USPSTF: U.S. Preventive Services Task Force
